# Comparison of Colour Stability Between Various Denture Base Resins on Staining and Denture Cleansing Using Commercially Available Denture Cleansers

**DOI:** 10.7759/cureus.6698

**Published:** 2020-01-19

**Authors:** Fathima Banu, Karthigeyan Jeyapalan, Anand Kumar V, Kajal Modi

**Affiliations:** 1 Prosthodontics, Faculty of Dental Sciences, Sri Ramachandra Institute of Higher Education and Research, Chennai, IND; 2 Dentistry, Faculty of Dental Sciences, Sri Ramachandra Institute of Higher Education and Research, Chennai, IND

**Keywords:** color stability, denture base resin, staining agent, denture cleanser

## Abstract

Statement of problem

Discoloration of denture base materials on routine usage has been one of the common esthetic problems encountered by patients.

Aim

To determine the color stability of three different denture base materials upon staining with beverages and denture cleansing using commercially available denture cleansers.

Materials and method

Three denture base materials were used for the study, processed according to the manufacturer’s recommendation, and cut into 40 study samples measuring approximately 1 cm x 1 cm with 1 mm thickness. The samples were divided into two groups, Group A and Group B, and spectrophotometric analysis of all samples was done to evaluate the base value of the color for comparative analysis. Group A was immersed in coffee and Group B was immersed in cola and color change was noted after 12 hours and then after 24 hours. The samples were then cleansed using a denture cleanser and analyzed again. All values were tabulated and statistically analyzed.

Result

On descriptive statistical analysis, polymethylmethacrylate (PMMA) had higher ∆E values at 12 hrs after immersion in coffee and cola; after 24 hrs, high impact PMMA had higher ∆E values in coffee and PMMA in cola. Two-way analysis of variance (ANOVA) analysis showed no statistically significant difference for the samples immersed in coffee, whereas samples immersed in cola at the end of 24 hrs showed a significant statistical difference.

Conclusion

Thermoplastic resin was the least staining denture base material when compared to conventional PMMA and high-impact PMMA when immersed in coffee and cola. There was no significant difference in the cleanability of all three-denture base materials.

## Introduction

A cast partial denture is the indicated mode of removable prosthodontic treatment modality, but it has been avoided in the recent past due to the display of metal causing an unaesthetic appearance. With increased aesthetic concerns, the use of alternative resin material, which could be more esthetic and functionally mimicking a cast partial denture base is needed. Though acrylic resins are commonly used, it is considered to be a temporary partial denture until a definitive prosthesis is fabricated. The use of thermoplastic resins and biofunctional prosthetic systems has tremendously increased along with polymethylmethacrylate (PMMA). The various materials available for fabricating dentures are chemically activated resin, heat-activated resin, thermoplastic resin, and high-impact resin. The chemically activated resins are commonly used for temporary dentures while the latter three are used for permanent denture fabrication. It is documented that PMMA, despite having favorable functional, physical, and mechanical properties, is more prone to discoloration with usage.

For the fabrication of non-metal clasp dentures, polyamide resins, polycarbonate resins, and polyethylene terephthalates were implemented as representative thermoplastic denture base resin products. Of these, polyamide resin, frequently known as nylon polymer, was launched as a base material for dentures in the 1950s. These polyamide resins are made from diamine and dibasic acid polymerization and have several benefits over standard PMMA, such as superior aesthetics and flexibility, claspless retention, and can also be used in conditions where the patient is allergic to monomer [1]. In contrast to heat-activated resin, the Biofunctional Prosthetic System (BPS) utilizes high-impact-resistant acrylic resin that is manufactured by replacing the PMMA in the powder with a styrene-butadiene copolymer and has been discovered to have increased impact strength and greater polishability [2].

The main disadvantage of the materials used for the fabrication of removable complete/partial dentures is the fact that their physical and chemical properties undergo rapid changes with time in the oral cavity. Color and translucency should be maintained during processing, and these resins should not get stained or change color in clinical use. The criteria for color stabilization may provide significant data about the serviceability of these products [3]. These color changes can occur due to intrinsic and extrinsic factors or a combination of both. Intrinsic factors, such as degree of conversion and presence of residual monomer, can influence color stability. Another possible source of color change is porosity, which can be caused due to overheating or variation in pressure during processing [4]. The extrinsic stain is time-dependent and associated with eating habits, such as consumption of tea, red wine, cola, and coffee, which act as an extrinsic factor for color alteration due to the absorption and adsorption of these stains [5-6]. The absorption and adhesion of the colorants deteriorate the quality of the material by causing surface roughness, accumulation of debris, and infection-causing organisms like Candida albicans, which can, in turn, lead to the damage of the underlying soft and hard tissues. The discoloration of materials used in the fabrication of these removable prostheses can lead to an unfavorable appearance and patient dissatisfaction. In addition, it reduces the quality of the prosthesis.

It’s been found that the magnitude of color change also depends on the maintenance of good oral hygiene and the frequency of using cleaning agents by patients [5,7]. Although changes are made in their composition to enhance their strength and polishability, it is still unclear whether these newer materials possess the same discoloration properties of conventional PMMA or they can maintain their color intraorally. The purpose of this study is to determine the color stability of three different denture base materials upon staining with two different colored liquids and the cleanability of the material using commercially available denture cleansers.

## Materials and methods

The study was conducted in vitro in the Department of Prosthodontics, Faculty of Dental Sciences, Sri Ramachandra Institute of Higher Education and Research (SRIHER). The denture base materials used for the study were conventional PMMA, thermoplastic resin, and high-impact denture base resin. The three materials were processed according to the manufacturer’s recommendation and were cut into 10 study samples for each material, measuring approximately 1 cm x 1 cm, with 1 mm thickness.

**Figure 1:**
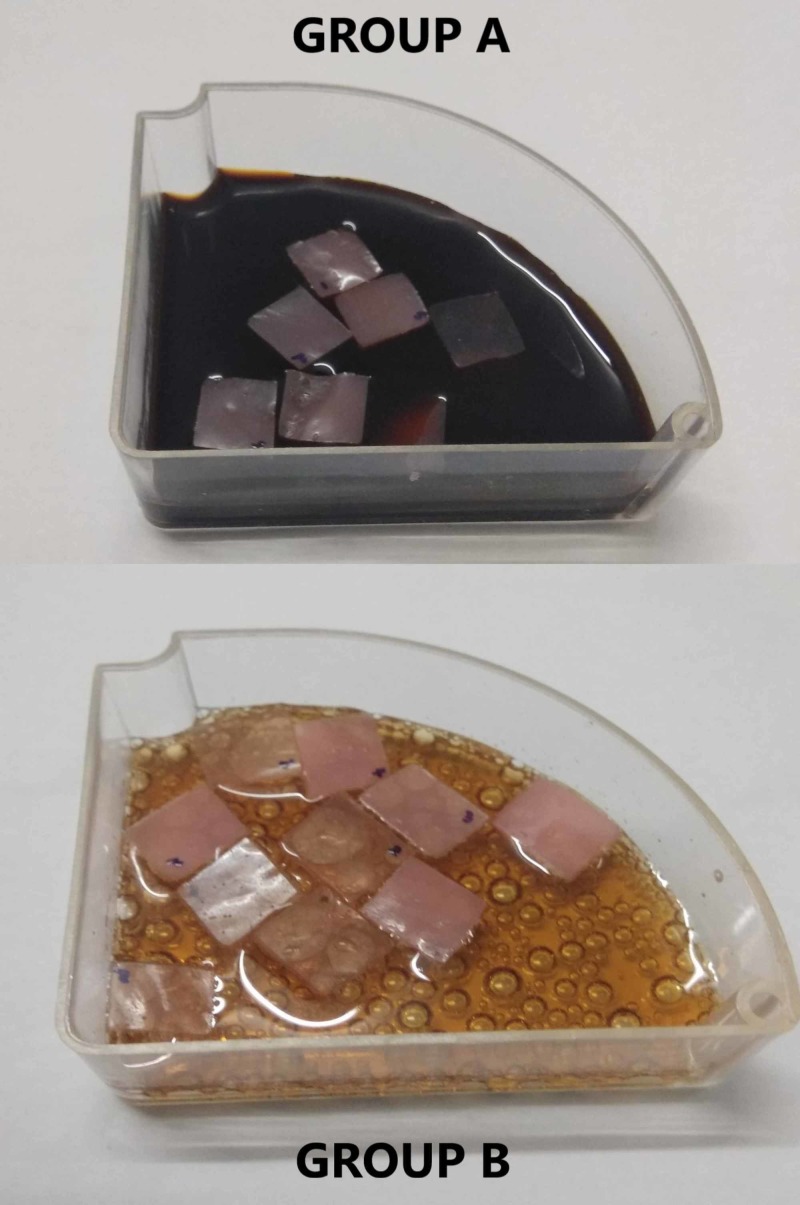
Staining agents used in the study

**Figure 2:**
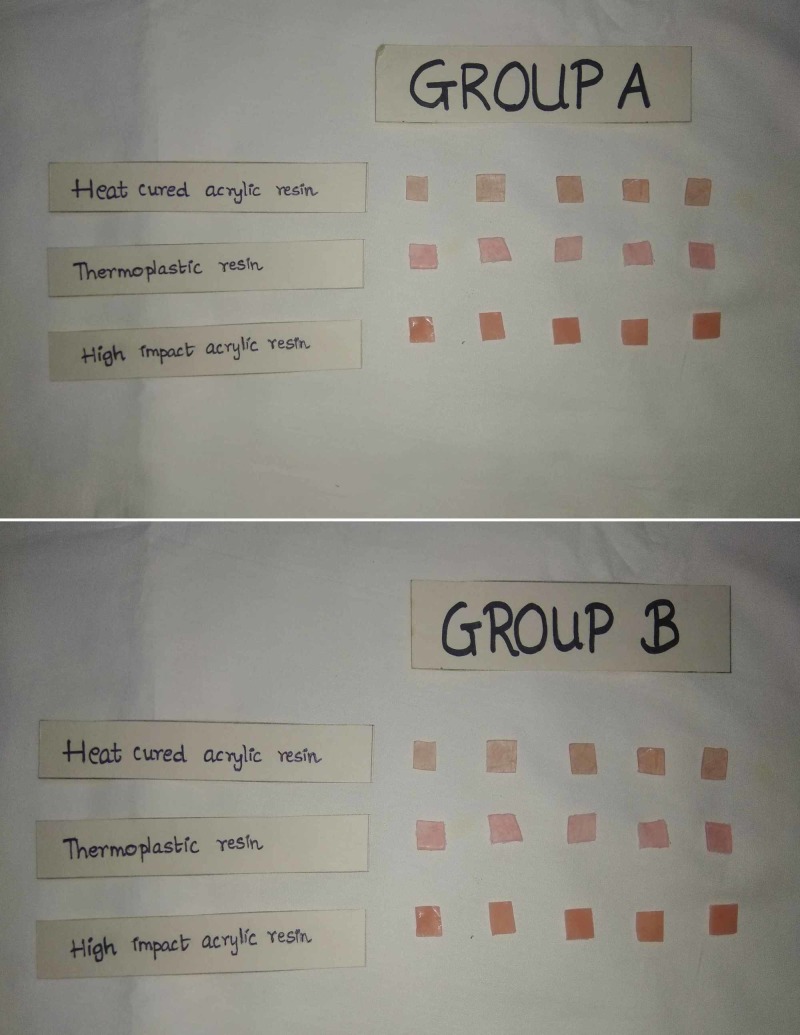
Samples after immersion in the staining agents

The samples were divided into two groups, Group A and Group B, each containing five samples of each type of material. A spectrophotometer analysis of all the samples was done to evaluate the base value of the color for comparative analysis. The spectrophotometer uses the CIE Lab system (International Commission on Illumination) that expresses color in numerical values. It determines the lightness (L), green-red component (a*), and blue-yellow component (b*). The magnitude of color difference can be obtained using the following formula: ∆E= √(L1-L2)2+ (a1-a2)2+(b1-b2)2. These values were assigned the variables BL, Ba, and Bb (B representing baseline value). The staining agents used in this study were coffee, a caffeinated beverage, and cola, an aerated beverage. Group A was immersed in coffee and Group B was immersed in cola (Figure 1). The values L, a, and b were checked at two time intervals, after 12 hours and after 24 hours. The values post 12 hours of staining were marked as L12, a12, and b12 and post 24 hours were L24, a24, and b24 (Figure 2).

The samples were then cleansed using a denture cleanser that contained sodium perborate and potassium persulphate as their active ingredients for 24 hours and the values L, a, and b were checked again and were marked as LC, aC, and bC. The magnitude of color changes was acquired using the mentioned formula. The ∆E12, ∆E24, and ∆EC values represent the magnitude of color difference between the prestained samples with the samples stained after 12 hours, 24 hours, and cleansing. The values obtained for all samples were tabulated and were statistically analyzed using SPSS software version 19.0 (IBM Corp., Armonk, NY).

## Results

On descriptive statistical analysis (Table 1), it was observed that in samples stained with coffee, PMMA was found to have higher ∆E values (5.3060) as compared to thermoplastic resin (3.0320) and high-impact PMMA (3.7280) at 12 hrs after immersion. After 24 hrs, high-impact PMMA was shown to have a higher ∆E value (5.6180) as compared to the other two samples. All three samples showed almost equal mean values after cleaning with a denture cleansing agent. Conventional PMMA showed higher ∆E values after immersion in cola at both 12 hrs (5.2460) and 24 hrs (3.3940) when compared to the other two samples. After treating with denture cleanser, PMMA showed a higher ∆E value (4.2520) than thermoplastic resin and high-impact PMMA.

**Table 1:** Descriptive statistical analysis ∆E12, ∆E24, and ∆EC represent the magnitude of color difference between the pre-stained samples with the samples stained after 12 hours, 24 hours, and cleansing with denture cleanser, respectively.

GROUPS			N	Mean	Std. Deviation
GROUP A	ΔE12	POLYMETHYLMETHACRYLATE	5	5.306	1.81092
		HIGH IMPACT POLYMETHYLMETHACRYLATE	5	3.728	2.89021
		THERMOPLASTIC RESIN	5	3.032	2.88931
	ΔE24	POLYMETHYLMETHACRYLATE	5	2.74	1.51096
		HIGH IMPACT POLYMETHYLMETHACRYLATE	5	5.618	3.93651
		THERMOPLASTIC RESIN	5	4.926	2.94333
	ΔEC	POLYMETHYLMETHACRYLATE	5	4.52	0.84086
		HIGH IMPACT POLYMETHYLMETHACRYLATE	5	4.408	0.89407
		THERMOPLASTIC RESIN	5	4.944	0.22568
GROUP B	ΔE12	POLYMETHYLMETHACRYLATE	5	5.246	1.79578
		HIGH IMPACT POLYMETHYLMETHACRYLATE	5	3.728	2.89021
		THERMOPLASTIC RESIN	5	3.032	2.88931
	ΔE24	POLYMETHYLMETHACRYLATE	5	3.394	0.81338
		HIGH IMPACT POLYMETHYLMETHACRYLATE	5	2.962	0.86173
		THERMOPLASTIC RESIN	5	1.76	0.92898
	ΔEC	POLYMETHYLMETHACRYLATE	5	4.252	0.77574
		HIGH IMPACT POLYMETHACRYLATE	5	3.51	0.40963
		THERMOPLASTIC RESIN	5	1.906	0.52885

Two-way ANOVA analysis (Table 2) showed no statistically significant difference for the samples immersed in coffee, whereas samples immersed in cola at the end of 24 hrs showed a significant statistical difference between the three groups (p≤0.05). In addition, a high statistical significance between the three groups was observed for samples treated with denture cleanser after immersion in cola (p≤0.01). Post-hoc Tukey analysis (Table 3) for the three groups revealed samples immersed in cola at the end of 24 hrs showed a significant difference between conventional PMMA and thermoplastic resin (p≤0.05), and between high-impact PMMA and thermoplastic resin (p≤0.05). A highly statistical significance was observed after cleansing for samples immersed in cola between conventional PMMA and thermoplastic resin (p≤0.01) and between high-impact PMMA and thermoplastic resin (p≤0.01).

**Table 2:** Two-way ANOVA ∆E12, ∆E24, and ∆EC represent the magnitude of the color difference between the prestained samples with the samples stained after 12 hours, 24 hours, and cleansing with denture cleanser, respectively. *Significant level at p<0.05

			Sig.
GROUP A	ΔE12	Between Groups	0.39
		Within Groups	
		Total	
	ΔE24	Between Groups	0.313
		Within Groups	
		Total	
	ΔEC	Between Groups	0.485
		Within Groups	
		Total	
GROUP B	ΔE12	Between Groups	0.409
		Within Groups	
		Total	
	ΔE24	Between Groups	0.03*
		Within Groups	
		Total	
	ΔEC	Between Groups	0*
		Within Groups	
		Total	

**Table 3:** Post-hoc intergroup comparison ∆E12, ∆E24, and ∆EC represent the magnitude of color difference between the prestained samples with the samples stained after 12 hours, 24 hours, and cleansing with denture cleanser, respectively. PMMA: polymethyl methacrylate; *Significant level at p<0.05

			Mean Difference (I-J)	Std. Error	Sig.
GROUP A	ΔE12	PMMA - HIGH IMPACT PMMA	1.578	1.63221	0.353
		PMMA-THERMOPLASTIC RESIN	2.274	1.63221	0.189
		HIGH IMPACT PMMA-THERMOPLASTIC RESIN	0.696	1.63221	0.677
	ΔE24	PMMA - HIGH IMPACT PMMA	-2.878	1.87767	0.151
		PMMA-THERMOPLASTIC RESIN	-2.186	1.87767	0.267
		HIGH IMPACT PMMA-THERMOPLASTIC RESIN	0.692	1.87767	0.719
	ΔEC	PMMA - HIGH IMPACT PMMA	0.112	0.45568	0.81
		PMMA-THERMOPLASTIC RESIN	-0.424	0.45568	0.37
		HIGH IMPACT PMMA-THERMOPLASTIC RESIN	-0.536	0.45568	0.262
GROUP B	ΔE12	PMMA - HIGH IMPACT PMMA	1.518	1.62998	0.37
		PMMA-THERMOPLASTIC RESIN	2.214	1.62998	0.199
		HIGH IMPACT PMMA-THERMOPLASTIC RESIN	0.696	1.62998	0.677
	ΔE24	PMMA - HIGH IMPACT PMMA	0.432	0.54981	0.447
		PMMA-THERMOPLASTIC RESIN	1.63400	0.54981	0.012*
		HIGH IMPACT PMMA-THERMOPLASTIC RESIN	1.20200	0.54981	0.049*
	ΔEC	PMMA - HIGH IMPACT PMMA	0.742	0.37403	0.071*
		PMMA-THERMOPLASTIC RESIN	2.34600	0.37403	0*
		HIGH IMPACT PMMA-THERMOPLASTIC RESIN	1.60400	0.37403	0.001*

## Discussion

Color instability is one of the most prevalent acrylic resin-related problems that can lead to complications of aesthetics and hygiene. Acrylic resins are susceptible to sorption owing to the method of absorption and adsorption of different colors [8]. In the past years, there have been several studies showing the comparisons in color stability of a different brand of artificial teeth between heat cured and chemically cured resins and even between different brands of heat-cured resins. It is of prime importance to evaluate which type of permanent denture base material is best suitable for the fabrication of prosthesis and in terms of its longevity based on color stability. Hence, a comparison between PMMA, thermoplastic resin, and high-impact PMMA (BPS) was made in the study. Various studies available have compared only their physical property but studies based on color stability, which is foremost important for the maintenance of denture hygiene and esthetic is still lacking.

Denture base material used for the fabrication of dentures has to be present in an atmosphere having variations in oral temperature, the pH of saliva, and their constituents and must be in contact with multiple foods and drinks taken at extreme temperatures that make them vulnerable to changes in their physical structure and appearance owing to the absorption of different contaminants [9]. Acrylic resin constitutes organic materials and its translucency and color are likely to deteriorate due to the adhesion of colorants to the surface pellicle layer forming on the denture base material when they come into contact with different compounds in food products and beverages.

Among them, coffee and soft drinks are the second and fifth-most commonly consumed beverage, respectively. Tannin and Caramel E150d are the chromogens responsible for the beverage color as well as for the discoloration of the denture base material. The present study utilized coffee and cola in which samples were immersed for a duration of 12 hrs and 24 hrs, representing 15 days and one month of daily consumption of the beverage, respectively, according to the literature support [10].

A spectrophotometer analysis of all the samples was done to evaluate the base value of the color for comparative analysis. The samples were divided into two groups, Group A representing samples immersed in coffee and Group B representing cola. To measure the changes in color, the spectrophotometer is a better option when compared with the Munsell color order, as instrumental measurement eliminates the subjective interpretation of visual color comparison [11]. The use of the uniform color scale of CIE Lab has the advantage of having its arrangement in an approximately uniform three-dimensional color space whose elements are spaced equally on the basis of parameters of visual color perception.

The stained samples after 12 hrs and 24 hrs evaluation were then cleansed using a denture cleanser to evaluate their ability to retain their original color even after subjecting them to different food colorants. While the most commonly employed method of denture cleaning appears to be the use of soap and brush, standard alkaline peroxide soak denture cleaners are used much more commonly than other denture cleaners that help them keep the dentures clean and devoid of any deposits [12]. Hence, a denture cleaner that contained sodium perborate and potassium persulphate, as their active ingredients were employed in the study and the samples were immersed in them for 24 hours and the values L, a and b were checked again. All the values collected by the spectrophotometric analysis of all the three different denture base materials were analyzed statistically for both the staining agents and for the cleansing agent. It was observed that there was no statistical difference between the three materials immersed in coffee while there was a numerical change in the E value with a mean value of more than 4.0.

Statistical analysis of the samples immersed in coffee showed no significance at both time intervals and after subjecting them to cleansers, suggesting that all three denture base materials behaved almost similar in getting stained and maintaining similar color after immersion in the denture cleanser solution. Though there was no statistical difference, the mean value of staining of all the three-denture base materials was more than four units. Samples immersed in cola didn’t have any statistically significant change at the 12 hrs interval but did show significance at the 24 hrs interval and after immersion in the denture cleanser. In addition, the mean value of E value ranged between 3 and 5.

Previous studies of coffee and tea staining of denture base products showed that samples immersed in coffee stained more because their brown pigmentation has lower polarity than that of green tea [13-14]. Another research noted that the change in color caused by coffee is more intense because it includes complex interactions between adsorption and absorption, facilitated by the affinity between colors and materials, whereas the change in color caused by green tea is mainly due to adsorption [15]. In accordance with this finding, all samples immersed in coffee showed more intense changes in color than those immersed in cola.

Among the denture base materials evaluated in the present study, thermoplastic resin showed better color stability when compared to conventional PMMA and high-impact PMMA at both the 24 hrs interval and after immersion in the denture cleanser. An E value of 5.2 obtained at 12 hrs for PMMA was found to be higher as compared to high-impact resin, whereas it was the least for thermoplastic resin. In dentistry, a discoloration that is more than perceptible (∆E >1.0) is considered acceptable up to a ∆E value of 3.3, above which it is considered unacceptable [16-17]. In general, heat-polymerized resins may be more susceptible to pigmentation than thermoplastic resins because heat-polymerized resins have a higher porosity affected by the techniques of processing and finishing [1]. In a study conducted by Mousavi S et al. on acrylic teeth, cola caused a relatively small change in the color as compared to coffee and tea [18]. This was in consensus with the present study, and this insignificant color change has been attributed to its relatively low pH (about 2 according to the manufacturer) [19]. Cola takes its color of light yellow to dark brown from the caramel color additive, which is prepared by heating sugar or glucose in the presence of a mineral or alkalic acid. Although cola had the lowest pH and might damage the surface integrity of the materials, it did not produce as much staining as red wine and coffee, and this was proven in studies conducted by Sepulveda-Navarro et al. [15], which was consistent with the results obtained in the current study. In addition to the staining comparison, the present study revealed thermoplastic resin to be stain-resistant as compared to the PMMA and high-impact resin.

The commercially available denture cleansers usually have a combination of ingredients, including oxidizing, effervescing, and chelating agents along with detergents and enzymes. Perborates are one of the most commonly used oxidizing (bleaching) agents for stain removal. The results obtained in the present study showed that ∆E values for all the samples were greater in samples subjected to denture cleanser after immersion in coffee, whereas for samples immersed in cola, thermoplastic resin showed the least change in color in both staining and cleansing agent. Jagger et al. in their study of the efficacy of denture cleaners on the removal of tea stain from PMMA resin found that irregularities and porosities on the denture surface played a significant part in the retention of stain and microbial plaque [20]. The mode of plaque attachment and stain build-up may be related to the roughness and porosity of the surface. These surface defects initiate plaque formation by protecting the bacteria from dislodging and may render complete removal of plaque and stain difficult. In high-impact resins, reinforcing fibers including veined fibers are incorporated to improve the strength. These reinforcing fibers impart difficulty in finishing and polishing, and the surface shows more microscopic irregularities than conventional resins [21]. The results obtained in this study too showed that conventional PMMA and high-impact PMMA had lesser cleanability than thermoplastic resin when they were subjected to conventional denture cleansers.

## Conclusions

Within the limitations of this study it was found that (1) thermoplastic resin was the least staining denture base material as compared to conventional PMMA and high-impact PMMA when immersed in coffee and cola; (2) there was no significant difference in the cleanability of all three denture base materials after subjecting them to a conventional denture cleanser when immersed in coffee; and (3) thermoplastic resin immersed in cola showed better cleanability when compared to the other two denture base materials. Although it was found that thermoplastic resin exhibited better color stability, more studies are needed comparing their mechanical properties with the physical properties of all the three denture base materials.
